# Pedestrian Trajectory Prediction for Real-Time Autonomous Systems via Context-Augmented Transformer Networks

**DOI:** 10.3390/s22197495

**Published:** 2022-10-02

**Authors:** Khaled Saleh

**Affiliations:** School of Information and Physical Sciences, The University of Newcastle, Callaghan, NSW 2308, Australia; khaled.saleh@newcastle.edu.au

**Keywords:** pedestrian, trajectory, intent, autonomous vehicles

## Abstract

Forecasting the trajectory of pedestrians in shared urban traffic environments from non-invasive sensor modalities is still considered one of the challenging problems facing the development of autonomous vehicles (AVs). In the literature, this problem is often tackled using recurrent neural networks (RNNs). Despite the powerful capabilities of RNNs in capturing the temporal dependency in the pedestrians’ motion trajectories, they were argued to be challenged when dealing with longer sequential data. Additionally, whilst the accommodation for contextual information (such as scene semantics and agents interactions) was shown to be effective for robust trajectory prediction, they can also impact the overall real-time performance of prediction system. Thus, in this work, we are introducing a framework based on the transformer networks that were demonstrated recently to be more efficient and outperformed RNNs in many sequential-based tasks. We relied on a fusion of sensor modalities, namely the past positional information, agent interactions information and scene physical semantics information as an input to our framework in order to not only provide a robust trajectory prediction of pedestrians, but also achieve real-time performance for multi-pedestrians’ trajectory prediction. We have evaluated our framework on three real-life datasets of pedestrians in shared urban traffic environments and it has outperformed the compared baseline approaches in both short-term and long-term prediction horizons. For the short-term prediction horizon, our approach has achieved lower scores according to the average displacement error and the root-mean squared error (ADE/RMSE) of predictions over the state-of-the art (SOTA) approach by more than 11 cm and 23 cm, respectively. While for the long-term prediction horizon, our approach has achieved lower ADE and FDE over the SOTA approach by more than 62 cm and 165 cm, respectively. Additionally, our approach has achieved superior real time performance by scoring only 0.025 s (i.e., it can provide 40 individual trajectory predictions per second).

## 1. Introduction

The interactions between pedestrians and highly/fully automated vehicles (AVs) requires a mutually shared understanding between the two parties [1]. On one hand, the vehicles need to have a deep understanding and anticipation of the pedestrians’ actions, especially in shared urban traffic environments. On the other hand, a suitable channel is also required so that the vehicles could convey their decisions to the pedestrians, similar to what typically happens between human-driven vehicles and pedestrians nowadays. Therefore, the problem of the trajectory prediction for pedestrians received the attention of the research community over the past few years. In literature, the formulation of the trajectory prediction of pedestrians problem is generally unified (i.e., given a short-time observation of the pedestrians’ motion trajectories, the task is to forecast a few seconds ahead the rest of their future trajectory). The setup and context of the problem, however, vary based on the target domain application. Target domain applications can be categorised into two main categories based on the nature of the space that with which the pedestrians are interacting. The first one is crowded spaces whether indoors [2,3] or outdoors [4,5,6,7,8,9,10], where a large number of pedestrians are close to each other and the interactions happen only among themselves (typically in hallways indoors or on side-walks outdoors). The second domain application is the urban traffic shared space, where the density of the pedestrians in the scene varies from medium to low and the interactions are more diversified (i.e., among each other, with cyclists and with vehicles) [11,12,13,14,15].

The focus of this work will be more on the second domain application, but we will also discuss some of the approaches from the first category, which overlap with our proposed methodology. In the literature, one of the most successful approaches for tackling the problem of the trajectory prediction of pedestrians (across the aforementioned two domain applications), are the data-driven approaches. The reason for that is the capability of such approaches to directly learn complex behaviours of pedestrians (especially in crowded spaces) in different scenarios using large datasets. One of the key components that commonly exist almost in all of the data-driven approaches is the recurrent neural networks (specifically Long Short-Term Memory (LSTM) architecture). LSTMs can implicitly model the inherent dependency between the consecutive observations of pedestrians’ trajectories in an end-to-end fashion. That being said, LSTMs were, however, recently argued to be inefficient when it comes to modelling longer sequential data [16]. Additionally, LSTMs were also shown to be more sensitive to missing observations, which is typically the case with any data coming from real physical sensors [17].

Thus, transformer networks [18] were recently introduced and quickly became the preferred model when it comes to sequential modelling tasks, such as natural language translation and summarisation [19]. The reason for that is the multi-head attention mechanism of these networks that allow them to process the sequential input data in parallel without any constraints on their order, as it is the case with LSTMs. Additionally, the attention mechanism allows the model to attend to different parts of the input sequence at each time-step, which in return provides a more in-depth reasoning about the relevancy and context of the input sequential data. Transformer networks were recently also explored for the trajectory prediction of the pedestrians’ problem in crowded spaces [17]. The model has achieved state-of-the-art (SOTA) results over the TrajNet benchmark [20]. The model was able to achieve such results by capitalizing only on the single positional information about the pedestrians in the scene. While positional information could be enough for the transformer model in crowded spaces, we argue that in urban traffic shared spaces this might not be the case. The rationale behind that is in crowded spaces, there is only one type of interactions (which is between pedestrians and each other).

On the contrary, in urban traffic shared spaces, there are two levels of interactions: the first one is the interaction between pedestrians and other agents sharing the space with them such as vehicles and cyclists. The second level of interaction happens between the pedestrians and the physical semantics of the scene such as trees, buildings, road, side-walk, vegetation, etc. While such semantic information might not be very useful in crowded spaces (since interactions take place only on side-walks), in urban traffic shared spaces, it might influence the decisions and choices of pedestrians while navigating through them. For example, pedestrians in urban traffic environments might cross the road or walk on grass instead of side-walks.

Thus, in this work, we are proposing an approach based on transformer networks, given their efficiency in capturing the temporal dependency in longer sequential data such as pedestrians’ trajectories, but rather than relying only on positional information as an input, we will be augmenting our proposed transformer network model with additional information about the context of the scene, namely interactions with other agents and semantics of the scene. In summary, the contribution of this work is four fold:

- A novel framework based on transformer networks that address the problem of pedestrians’ trajectory prediction in urban traffic shared spaces.
- An efficient representations of contextual information that account for the preference of pedestrians in urban traffic shared spaces.
- A scalable framework that can provide multi-pedestrians’ trajectory predictions (more than 40 per second) in real-time without compromise on accuracy.
- A robust data-driven approach that can generalise to unseen scenarios across different urban traffic shared spaces.

The rest of the paper will be organised as follows. In Section 2, a summary of the related work from the literature will be discussed. In Section 3, the proposed methodology will be presented and discussed. Section 4 will include the experimental results of our proposed approach on two real datasets. Finally, Section 5 will conclude our paper.

## 2. Related Work

The problem of the trajectory prediction of pedestrians is often tackled using two broad classes of approaches, namely model-based approaches and data-driven approaches. In model-based approaches, as the name implies, an explicit model about the pedestrian actions (in terms of movements over time) is assumed to be existent in the first place. Commonly, model-based approaches are also assumed to be holding the Markovian assumption (where the future state or position of pedestrians is only conditioned on their current state). Another unique feature of model-based approaches is that they implicitly can account for the uncertainties in the actions of rationale agents such as pedestrians [21].

In [22], several techniques based on linear dynamical motion models such as the Kalman filter and Gaussian process were introduced to predict future trajectories of pedestrians over short-term horizons (i.e., whether pedestrians will stop to cross the road or continue walking). To overcome the limitations of linear dynamical models, Karasev et al. [23], proposed another model-based approach by modelling pedestrians actions’ as a jump-Markov process for the long-term prediction of their trajectories. They utilised a Rao-Blackwellized filter to estimate the posterior over a switching goal state that pedestrians are heading towards, so that it could be changed over-time. Then, they cast the problem as a Markov decision process (MDP), which they solve using an estimated reward function based on a semantic map of the scene.

More recently, Anderson et al. [24], presented a model-based approach that models the interaction between pedestrians and incoming vehicles that they might encounter in urban traffic shared spaces. Given an *n* number of vehicles on the scene, they model the influence of each vehicle on each pedestrian’s decision whether he/she will yield to it using an attention mechanism. Their attention mechanism is hand-crafted using an assumed known position and velocity for each vehicle and pedestrian as well as a pre-defined threshold distance to discard any vehicle beyond this distance. Given this hand-crafted interaction modelling, they estimate the future trajectories of pedestrians using a constant velocity Kalman filter.

In data-driven approaches, conversely, there is no explicit modelling of the pedestrians’ behaviours. They, however, rely on datasets of pedestrian trajectories in a number of scenarios and try to learn the pedestrian behaviours directly from the data. In [25], they introduced one of the early techniques that relied on LSTM architecture for predicting pedestrians’ trajectories. The input to their LSTM model was only the 2D positional information of pedestrians that were observed over a window of 1 s using a vehicle-based stereo-camera. During the inference stage, they predict up to 4 s ahead of the future trajectories of the pedestrians in the scene. One of the relatively recent yet successful data-driven approaches is the Social-LSTM model introduced in [5]. The Social-LSTM model was targeted for the prediction of pedestrian trajectories in crowded spaces by exploiting the interactions between pedestrians on side-walks. They utilised LSTM networks to automatically model the sequential positional information of pedestrians. Moreover, their model included a social pooling layer to model potential social interactions among pedestrians within a pre-defined grid size around each pedestrian. Due to the computational complexity of the social pooling layer of the Social-LSTM model to take into account the influence of the interactions among all pedestrians in the scene, the Social-GAN model [6] was introduced to overcome this issue. The Social-GAN model addressed this limitation by relying on two main components; first, is the Generative Adversarial Networks (GANs), which are used to capture and generate more socially-acceptable trajectories. Second, they utilised a global pooling mechanism rather than the average layer used in the Social-LSTM model.

More recently, the number of data-driven approaches introduced for the pedestrian trajectory prediction problem were experimenting with graph neural networks either as a stand-alone framework [26] or through fusion with LSTM for better representation of input features to LSTM networks [27]. In [26], they proposed a graph convolutional neural network model that focuses on the interactions among pedestrians in crowded spaces by modelling them as a graph and embedding those social interactions as a kernel function as part of the adjacency matrix. More closely related work is by Salzmann et al. [27], where they proposed a graph-structured recurrent neural network model for pedestrians’ trajectory prediction that takes into account interactions between agents in urban traffic environments as well as the influence of physical environment via a semantic map of the scene. They represented pedestrians as nodes in graph and modelled interactions between them and different agents (pedestrians or motorists) via graph edges. Finally, in [17], another data-driven approach was proposed but instead of utilising the famous LSTM network, they, however, relied on the transformer networks that have been achieving SOTA results in several natural language processing tasks. They proposed a vanilla transformer network model that takes an input only positional information about individual pedestrians without taking into account any social/contextual interactions. Their proposed model was one of the top performing models (outperforming other LSTM-based approaches) on the TrajNet benchmark [20], which is a large collection of pedestrians trajectory datasets in versatile crowded spaces.

Based on the reviewed related works from both the model-based approaches and the data-driven approaches, we can notice the following. In model-based approaches, they generally tend to be more simpler to implement; however, they rely on prior information that are not necessarily easy to obtain, such as the motion model of pedestrians, which is not fixed and changes a lot over time. Moreover, model-based approaches are often challenged when it comes to accommodating contextual information such as scene semantics, which was demonstrated to be of a great influence on actions of pedestrians in shared urban traffic environments [12,14].

Therefore, over the past few years, data-driven approaches become more commonly utilised because they do not suffer from those challenges associated with model-based approaches. That being said, data-driven approaches introduced in the literature are still facing some challenges when it comes to longer prediction horizons and real-time performance. One of the main reasons for that, is because majority of data-driven approaches rely on RNNs/LSTMs, which were shown in the literature to be inefficient when it comes to modelling longer sequential data [16]. Additionally, because of the complex representations of contextual information, such as graph-based representations [27], which require additional computational resources that affect the overall run-time performance of the traditional data-driven approaches, they are commonly introduced in the literature.

Thus, in our work we tried to overcome the aforementioned challenges with existing data-driven approaches from the literature by proposing a robust architecture and unique framework (which will be discussed in detail in the following section) based on transformer networks that can provide more accurate longer term prediction of pedestrians’ future trajectories when compared with RNN/LSTM and graph-based approaches without sacrificing the real-time performance, which is crucial for the reliable operation of AVs.

## 3. Proposed Method

**Figure 1:**
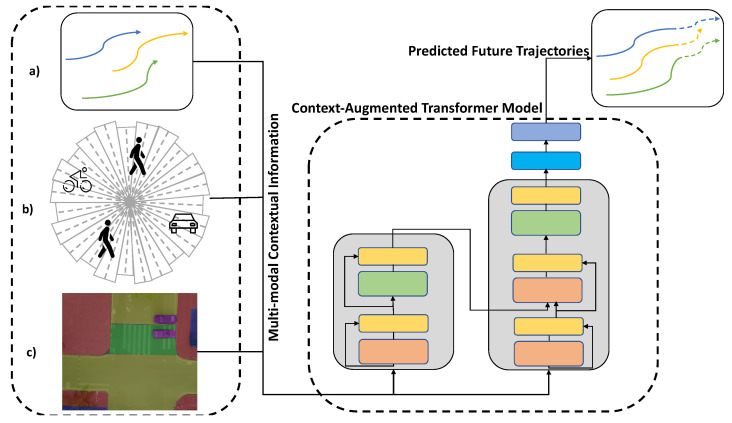
Our proposed context-augmented transformer framework for pedestrians’ trajectory prediction. The input is a multimodal contextual information: (**a**) past observed positional information, (**b**) agent interactions information and (**c**) scene semantics information. The output from our framework is the future positional information of all pedestrians in the scene.

In this section, we first start with our formulation for the trajectory prediction problem. Then, we present the different contextual information we took into account as an input to our proposed framework (shown in Figure 1). Then, we describe the architecture of our proposed context-augmented transformer model and its implementation details.

### 3.1. Problem Formulation

We cast the pedestrians’ trajectory prediction problem as a sequence prediction problem, where given past short-term observations about the pedestrians’ state $X_{t-\delta :t}$ at current time-step *t*, the task is to provide predictions about the true position of their future trajectory κ steps ahead. The state of pedestrians at each time-step can be approximated using multi-modal contextual information, which, in our case, are as follows.

#### 3.1.1. Positional Information

Given the bird’s eye view of the scene, we obtain the sequence of 2D positions (x, y) in the ground plane of each pedestrian in the scene. Then, we calculate the offset positions between every two consecutive positions and use it as our first dimension of the state representation input to our context-augmented transformer model. The reason for using offset positions instead of absolute positions is to make our trained model agnostic to the size dimensionality of different scenes views. Additionally, the position offsets can be viewed as an approximation of the pedestrians’ velocities over time, which could provide some patterns about pedestrians’ movements that can be captured by our transformer model.

#### 3.1.2. Agent Interactions Information

Similar to social interaction models [5,6], we also include interactions’ information as part of our pedestrians’ state representation. However, rather than having only interactions between pedestrians and each other, we also accommodate interactions with other agents such as moving vehicles and cyclists, which are commonly found in urban traffic shared spaces. The interactions information is included, using a polar occupancy grid map around each pedestrian in the scene, in which its centroid is the pedestrian’s 2D position. Encoding agent interactions as a polar occupancy grid map have been commonly applied in modelling pedestrians interactions in crowded spaces [12,28]. Each cell within a polar grid is parametrised by two parameters; namely, the orientation angle θ and radius *r*. *r* represents the distance from each agent in the scene and the ego-pedestrian position (i.e., centroid of the polar grid). Meanwhile, θ describes the orientation angle from each agent in the scene and the ego-pedestrian position. At each time-step *t* of the ego-pedestrian trajectory, if the euclidean distance between it and each agent is within a certain threshold th, then all those agents will be added to the cell (r,θ) of the polar grid interaction map of the ego pedestrian.

#### 3.1.3. Scene Semantics Information

The last information we take into account as part of the pedestrians’ state representation is the scene of semantic information. Given the bird’s eye view RGB image of the scene, we obtain the label map of each pixel that belongs to any class of the following five classes:

(1) road;
(2) side-walk;
(3) zebra-crossing;
(4) vegetation/grass;
(5) parked vehicle.

We focused on these specific five classes, since they are considered the most important ones that could influence the decisions/actions of pedestrians on the scene. For example, if a pedestrian moving on the road is encountered by a parked car, s/he would follow a trajectory that avoids colliding with it. The same also happens if there is a zebra crossing in the road; pedestrians would most likely cross through it if they want to reach the other side of the road. The way we accommodate the scene semantic information is by aggregating *k*-nearest neighbour pixel-wise semantics within a certain threshold distance (in pixels) at each time-step *t* along the 2D positions of the ego-pedestrian’s trajectory.

### 3.2. Context-Augmented Transformer Model

Given the advantages mentioned previously about the transformer networks model over RNN/LSTM networks, we are proposing a transformer model similar to the one proposed by Guliari et al., in [17], which achieved SOTA results on the leading benchmark for pedestrian trajectory prediction in crowded spaces, TrajNet [20]. Unlike their model, however, instead of using only positional information, we will be utilising the full contextual information described in the previous section fused together via a concatenation operation; hence, we refer to it as the context-augmented transformer model. We argue that including such contextual information besides the positional information will help in boosting the performance of our proposed transformer model, especially in urban traffic shared spaces. The overall architecture of the transformer model is following the same encoder/decoder paradigm that commonly exists in LSTM-based approaches. However, it is more efficient, as it does not contain the recurrences loops that exist in LSTM networks.

**Figure 2:**
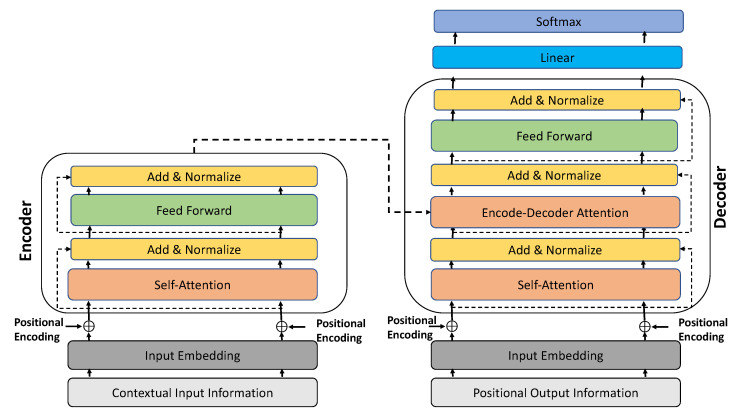
The building blocks of our proposed context-augmented transformer model.

The main building blocks of our context-augmented transformer model are the same as the original transformer model first introduced in [18]. These blocks namely are embedding layers, positional encoding layers, self-attention mechanism and fully-connected feedforward layers. The embedding layers exist at the start of both the encoder and the decoder stages, as shown in Figure 2. They are responsible for embedding both our observed (source) contextual sequence information and the target pedestrian’s trajectory into a higher dimensional space $d_{model}$. The embedding layers are essentially learnable linear transformations with weight matrices. Similar to embedding layers, positional encoding layers also are found at the start of the encoder/decoder stages. Positional encoding layers play a crucial role, given the fact that the transformer network model does not contain any recurrences, such as LSTMs. Thus, positional encoder layers give the transformer model the notion of order in the input/output sequential data (i.e. allows time-stamping). There are a number of pathways to define positional encoding; in our model, we follow the formulation introduced in [18]. In this formulation, assuming we have a sequence of length *L* and and we require the position of the *p*-th object within this sequence, the position encoding PE is calculated using a wide spectrum of frequencies of sine/cosine functions, as follows:

(1) $$\begin{array}{c} PE_{\left(p,2k\right)}=sin\left(p/{10,000}^{2k/d_{model}}\right) \end{array}$$

(2) $$\begin{array}{c} PE_{\left(p,2k+1\right)}=cos\left(p/{10,000}^{2k/d_{model}}\right) \end{array}$$

where *p* represents the position of an object in the input sequence (0≤p<L/2), and *k* is used for mapping to column indices ($0\leq k<d_{model}/2$). A single value of *k* maps to both sine and cosine functions. From the above formulation, we can observe that even positions correspond to the sine function and odd positions correspond to the cosine function. We can also deduce that for each dimension *k* of PE vector, it has a corresponding sinusoid that spans a frequency range from 2π to 10,000·2π. In other words, this will allow the model to be mindful of the order in the sequential data using unique relative positions. The dimension of the PE vector is similar to the embedding layers dimension, which is $d_{model}$ (since they are summed together, as shown in Figure 2).

Overall, the encoder/decoder parts of our context-augmented transformer model contain six layers in total. Internally, each layer is composed of both self-attention head and feed-forward fully connected sub-layers. Additionally, each sub-layer is followed by two residual connections and a normalisation operation. The multi-head self-attention, or the multi-scaled dot-product attention, works based on the mapping between the so-called ‘query’ vectors and the pair (key, value) vectors. The dimension of query and key vectors is $d_{k}$, whereas the values vector dimension is $d_{v}$. The attention operation itself is computed by taking the dot-product between the query and key vectors divided by the square root of $d_{k}$, before finally passing them to the Softmax function to obtain their weights by their values. Since the scaled dot-product attention operation is conducted multiple times in parallel, the queries, keys and values vectors are extended into matrices Q, K, V, respectively. The following formula is the description of how the scaled dot-product attention operation is calculated:

(3) $$\mathrm{Attention}\left(Q,\;K,\;V\right)=\mathrm{softmax}\left(\frac{QK^{T}}{\sqrt{d_{k}}}\right)V\;$$

Based on the previous equation, we can notice that the output of the scaled dotproduct attention operation can be viewed as a weighted sum of the values, where the weight assigned to each value is determined by the dot-product of the query with all the keys.

At the encoder stage, the embedding input is encoded and gives as an output of the queries, keys and values matrices calculated in Equation (3). These matrices are then passed to the decoder stage, which, in return at each prediction step, compares its own new queries matrix with the encoder’s keys and values matrices as well as its preceding decoded prediction. The decoder auto-regressively repeats the aforementioned procedure until all of the to be predicted total trajectory positions are achieved.

In this section, we will first present the datasets we utilised for training and evaluating the performance of our proposed approach. Then, the details of the setup of our experiment will be outlined. Finally, the quantitative and qualitative results of our proposed approach on real-life datasets will be evaluated and discussed.

### 3.3. Datasets

**Figure 3:**
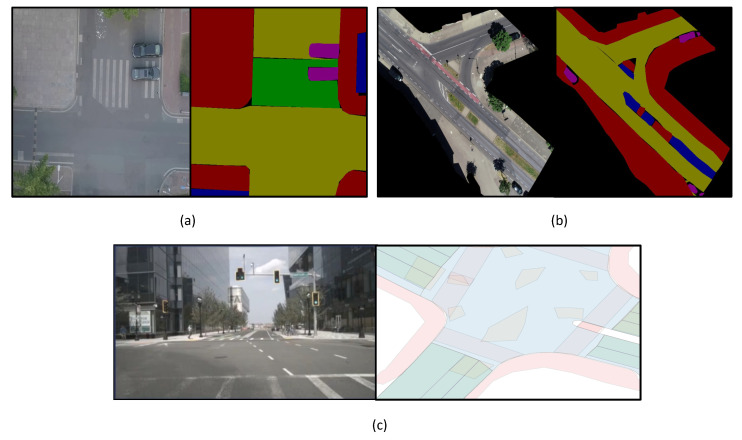
Sample images of an urban traffic shared space with its corresponding semantic label map from (**a**) DUT dataset [29], (**b**) inD dataset [30] and (**c**) nuScenes dataset [31].

The number of publicly available datasets of pedestrians trajectories in urban traffic shared spaces (interacting agents such as vehicles, cyclists… etc.) is rather limited. Just recently, three promising datasets became available, namely DUT [29], inD [30] and nuScenes [31]. The DUT dataset (shown in Figure 3a) contains two unique scenes of urban traffic shared spaces where pedestrians and moving vehicles are interacting with each other. The dataset has more than 1700 pedestrians’ trajectories that were captured by a drone with a down-facing camera hovering over a university campus. The captured video files were captured at 23.98 frame-per-second (FPS) and have a spatial dimension of 1920 H × 1080 W. The annotation in the dataset was done semi-automatically using a visual object tracker to extract pedestrians and vehicle trajectories with manual refinement if needed. The annotation contains the 2D position and velocity of pedestrians and vehicles both in pixels and meters. The inD dataset (shown in Figure 3b), contains more trajectories with roughly 11,500 different road users such as pedestrians, vehicles and bicyclists and it was captured also using a hovering drone with a down-facing camera. The inD dataset has a total recording time of 10 h and it was covering four intersections. The resolution of the recorded video files is 4096 H × 2160 W and captured at 25 FPS. Similar to DUT, the inD dataset was also annotated with 2D positions and velocity for both pedestrians and vehicles and unlike the DUT dataset, it also has the annotation for the bicyclists in the scene.

## 4. Experiments and Results

Finally, the nuScenes dataset [31], one of the largest autonomous vehicles datasets for many perception tasks recently provided both vehicles’ and pedestrians’ trajectories for the task of motion prediction. The nuScenes’ trajectory prediction dataset contains unique 700 urban traffic environment scenes for training with roughly 115 K pedestrians’ trajectories and another 150 scenes for validation with roughly 21 K pedestrians’ trajectories. The sampling rate for the dataset is 2 Hz and on average each pedestrian’s trajectory length ranges between 7–8 s.

### 4.1. Implementation Details

The first step in our experiments is to pre-process the datasets to obtain the required multi-modal input information discussed in Section 3.1. Since the FPS/sampling rate for each dataset of the aforementioned three datasets is not unified, for the DUT and inD datasets we down-sampled them to 10 FPS. On the other hand, for the nuScenes, we did not down-sample it because it already has a lower frame rate at 2 Hz. Given the formulation we discussed in Section 3.1, we slide a window of length δ+κ over each pedestrian’s trajectory from the three datasets. To conform with other approaches from the literature, during the training phase for the DUT and inD datasets we chose the size of the observed trajectory δ to be of size 30 (i.e., 3 s) and the size of the prediction horizon κ to be 50 (i.e., 5 s ahead). While for the nuScenes, we chose the size of the observed trajectory δ to be of size 8 (i.e., 4 s) and the size of the prediction horizon κ to be 6 (i.e., 3 s ahead).

For the positional information, we directly utilised the annotated 2D positions provided for each dataset. Since we are using offset positions instead of absolute positions as the input to our proposed transformer model, we subtracted each position from its preceding one for each pedestrian after the first observed 2D position. For the scene semantic information, we manually annotated the total 60 BEV images of the scenes from the DUT and inD datasets (28 for the DUT and 32 for the inD). The five labelled classes are shown in Figure 3. For the nuScenes, each scene contains a rasterized semantic map shown in Figure 3. Regarding the agent interaction information, in the DUT and inD datasets, we chose the parameters of the polar grid occupancy to be 64 pixels for the threshold value th of the euclidean distance between ego-pedestrian and all other agents in the scene. Meanwhile, for the nuScenes, we set the threshold value th to 10 and 20 m between the ego-pedestrian and the other pedestrians and other vehicles, respectively, similar to [27].

Given this pre-processed input multimodal contextual information, we trained our context-augmented transformer model. Since our formulation of the problem of pedestrian trajectory prediction is considered a regression problem, we chose the L2-loss function as our objective function for the training. We trained our model for 250 epochs using the Adam optimiser. For the hyper-parameters of the model, we chose $d_{model}$ to be 512 and we utilised 8 self-attention heads in both the encoder and decoder stages.

### 4.2. Performance Evaluation

We relied on the three datasets described above to evaluate the performance of our proposed framework. In order to do so, we will carry out two sets of experiments. Since the DUT and the inD datasets are quite similar to each other, we will rely on them in our first experiment, while the nuScenes dataset will be utilised for our second experiment. The objective of our first experiment is to validate our two stated hypotheses in Section 1. The two hypotheses were:

(1) Transformer-based approaches are more resilient than LSTM-based ones when it comes to long pedestrians’ trajectory prediction;
(2) The added contextual information to our proposed context-augmented transformer network will provide more accurate predictions about pedestrians’ trajectories when compared to the vanilla transformer network and other baseline approaches that utilise only positional information.

For our second experiment, the objective is to compare the performance of our proposed approach against SOTA techniques that natively, as part of its architecture, take into account the same three types of input features we discussed in Section 3.1 (namely positional information, agent interactions and scene semantics).

#### 4.2.1. DUT and inD Results

To that end, for our first experiment and to validate the first aforementioned hypothesis, we have trained an LSTM-based approach that utilises an encoder-decoder architecture similar to our proposed framework. The trained LSTM model is similar to the vanilla LSTM model proposed in [32], where both the encoder/decoder consists of one embedding layer and one LSTM layer and ends with one linear output layer. The embedding layers have a size of 64, and the encoder’s LSTM layer has 64 hidden units, while the decoder’s LSTM layer has 128 hidden units. We have trained two variants of this LSTM model, where one variant utilises only the positional information as input and we refer to it as ‘Vanilla-LSTM’. Meanwhile, the other variant utilises the same contextual information we utilised in our proposed context-augmented transformer network framework in addition to the pedestrians’ positional information. We refer to this model as ‘Context-LSTM’. For the validation of our second hypothesis, we have trained the transformer model introduced in [17], which only takes into account the positional information, unlike our proposed context-augmented transformer model. This model was one of the top-ranked models in the TrajNet challenge benchmark. We refer to this model as the ‘Vanilla-TF’.

The strategy we followed for the training and testing of the aforementioned models is similar to the one in [24]. The strategy is to train each model (including our proposed model) on one dataset from the DUT and InD datasets and test it on the other unseen dataset. The evaluation metrics we utilised to quantitatively assess the performance of our proposed model and all other compared models are two, namely the Average Displacement Error (ADE) and Root Mean Squared Error (RMSE) [33]. The reason we chose these two especially is that they are the most commonly used evaluation metrics when it comes to evaluating pedestrians’ trajectories, especially in urban traffic shared spaces [5,6,11,34]. ADE measures the expected distance (Euclidean) between the predicted position at each time step of the trajectory and the ground-truth position at that step according to the following formula:

(4) $$ADE\left(t\right)=\frac{1}{N}\sum_{i=1}^{N}E\left[{∥x_{i,t}-{\hat{x}}_{i,t}∥}_{2}\right]$$

where $x_{i,t}$ is the ground-truth position at time-step *t* of the trajectory *i*. Meanwhile, ${\hat{x}}_{i,t}$ is the predicted position at time-step *t* of the trajectory *i*.

In RMSE, it calculates the square root of the squared error between the predicted position at each time step of the trajectory and the ground-truth position at that step according to the following formula:

(5) $$RMSE\left(t\right)=\sqrt{\frac{1}{N}\sum_{i=1}^{N}E\left[{∥x_{i,t}-{\hat{x}}_{i,t}∥}_{2}^{2}\right]}$$

where $x_{i,t}$ is the ground-truth position at time-step *t* of the trajectory *i*. Meanwhile, ${\hat{x}}_{i,t}$ is the predicted position at time-step *t* of the trajectory *i*.

In Table 1, we report both the ADE and RMSE scores (in meters) of the aforementioned models on both the DUT and inD datasets over a prediction horizon of 5 s ahead. As can be noticed from the table, our first hypothesis was proved to be true, as both the transformer-based models (our ‘Context-TF’ and the ‘Vanilla-TF’) outperformed the LSTM-based models when tested on the two datasets with more than 0.19/0.05 improvement in ADE/RMSE scores. Furthermore, our second hypothesis as well was proved to be true as the added contextual information to both our ‘Context-TF’ model, and the ‘Context-LSTM’ model was shown to be helping in boosting the prediction capabilities of the models in terms of ADE/RMSE scores. More importantly, our ‘Context-TF’ model was shown to be the best performing model over the DUT and inD datasets with the lowest ADE/RMSE scores of 0.97/0.88 and 0.8/0.88, respectively.

In order to further investigate the influence of the individual contextual information on the performance of our proposed approach, we have trained three variants of our ‘Context-TF’ model. The first variant is utilising only the positional information, while the second variant is utilising the positional information and the agent interactions’ information (Context-TF${}_{INT}$); finally, the third variant is only utilising the positional information and the scene semantics information (Context-TF${}_{SEM}$). As it can be observed in Table 2, the individual variants on their own did not achieve better results than the combination of them both (as shown in the last row in Table 1), which further prove our hypothesis that the fusion of such contextual information is indeed beneficial when it comes to predicting the long-term trajectories of pedestrians in a shared urban traffic environment.

As it can be noticed also from Table 2, the interactions only model (Context-TF${}_{INT}$), tend to be achieving slightly better results on the DUT dataset in comparison to the semantics only model (Context-TF${}_{SEM}$). One of the main reasons for that is that the DUT dataset has more frequent interactions between pedestrians and other road-sharing users, since it was captured inside a university campus. Conversely, in the inD dataset, we can notice that the semantics only model (Context-TF${}_{SEM}$) scored slightly lower ADE/RMSE scores because of the fact that the inD dataset is larger in terms of the number of trajectories, and it was captured over extended areas of versatile urban traffic environments. Hence, it includes richer semantic information.

Furthermore, we compared the performance of our ‘Context-TF’ model against a number of baseline approaches from the literature. Similar to our first experiment, we trained each model on one dataset and tested it on the other unseen dataset. The results of our proposed model against the baseline model are shown in Table 3. The baseline models that we compared our proposed model against are four models (two data-driven approaches and the other two are model-based approaches). The first data-driven baseline approach is the Social-GAN model [6], which was one of the SOTA techniques for pedestrians’ trajectory prediction in crowded spaces. The second data-driven baseline approach is the Multi-Agent Tensor Fusion (MATF) [35] approach. MATF is based on the GAN architecture that utilises global average pooling layer to accommodate both interactions between pedestrians and the semantics of the environment. It is worth noting that the hyper-parameters for the two models, Social-GAN and MATF, are similar to the ones described in the original implementations in [6,35]. For the model-based baseline approaches, the first baseline is based on a linear dynamical motion model, where a constant velocity (CV) is assumed for the pedestrians, and hence we refer to this model as ‘Linear-CV’. The other baseline model-based approach we compared against is the model introduced in [24]. This model is referred to as the Off the Sidewalk Predictions (OSP), as it was denoted by its designers. OSP relies on the Kalman filter to predict future trajectories of pedestrians, and they take into account the interactions between pedestrians and vehicles using a hard-attention mechanism. The hyper-parameters for the OSP are the same as the best performing model on DUT/inD, as implemented in [24].

As it can be shown from Table 3, our proposed context-augmented transformer model ‘Context-TF’ has outperformed all the baseline models in terms of ADE/RMSE scores over both the DUT and the inD datasets. These scores further prove our claims that having more contextual information to the transformer model helped in boosting the overall performance of the model. Furthermore, in Table 3, we also report the performance of our proposed model with all the baseline models over short-term prediction horizons, namely 1, 2, 3 and 4 s ahead. Our context-augmented transformer model continued to provide robust results among the short-term prediction horizons; the only exception was in the DUT dataset, where the OSP and Vanilla-TF achieved lower ADE/RMSE scores over shorter time prediction horizons (1 and 2 s). We believe the reason for that, is that at such shorter time prediction horizons, there is not enough contextual information that could be discriminative enough to help in providing more accurate predictions. Moreover, the trajectories in the DUT dataset took place on a university campus where pedestrians might not be following the common-sense rules in urban traffic environments (such as pedestrians would generally prefer to walk on the side-walk). Additionally, another reason for that is the fact that OSP relies on a Kalman filter for giving predictions, which is well known for its competitive reasonable results in the shorter time prediction horizons [11,22]. On the contrary, the Social-GAN and MATF models have failed in giving competitive scores in comparison to our proposed model and the other baseline models. The reason for that is due to the fact that the number of interactions between pedestrians and each other in urban shared traffic spaces is not in line with the same quantity and density of the interactions that appear in crowded spaces.

**Figure 4:**
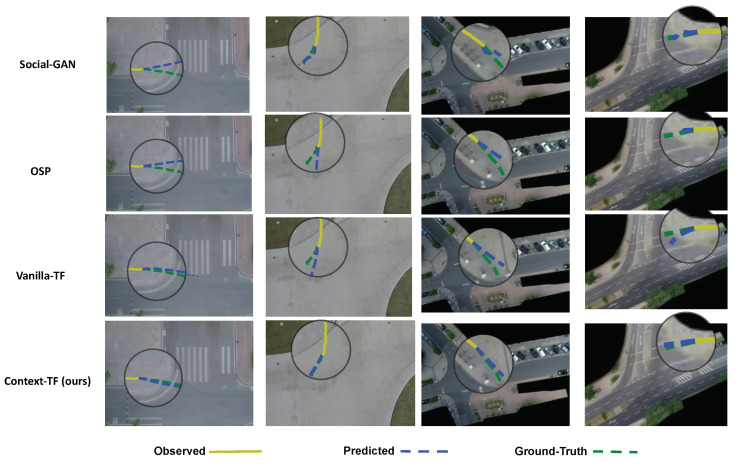
Sample output predictions of our proposed framework ‘Context-TF’ two different scenes from the DUT dataset (first two columns) and the inD dataset (second two columns). The observed short trajectory (3 s) is visualised in solid dark yellow, the predicted trajectory (5 s ahead) is in dashed blue and the ground-truth trajectory visualised in dashed green.

In order to further assess the performance of our proposed context-augmented transformer model, we qualitatively visualise some of the output predictions from our ‘Context-TF’ model along with the compared baseline models on two scenes from the DUT and inD datasets in Figure 4. The rows in Figure 4, correspond to the baseline compared models namely, Social-GAN, OSP, Vanilla-TF and our Context-TF, respectively. As it can be shown, our Context-TF model gives quite accurate predictions (in dashed blue), that, in the majority of the scenarios, are aligned with the ground-truth trajectory (in dashed green), given the observed short sequence (in solid dark yellow). Additionally, it can be noticed that our model can capture the non-linearity of the pedestrians’ trajectory and how it can respect the contextual information of the scene. For instance, in the second and fourth columns, we can observe how resilient is the predicted trajectory from our Context-TF model that follows the non-linear ground-truth trajectory. Meanwhile, we can notice how the other compared baseline models are challenged in most of the scenarios. For example, the Social-GAN, the OSP and the Vanilla-TF models in the scenario from the DUT dataset in the third column of Figure 4. We can see how the predictions from the three models diverge away from the ground-truth trajectory, while our Context-TF model provided more confident predictions.

In order to further assess the performance of our proposed context-augmented transformer model, we qualitatively visualise some of the output predictions from our ‘Context-TF’ model along with the compared baseline models on two scenes from the DUT and inD datasets in Figure 4. The rows in Figure 4, correspond to the baseline compared models namely, Social-GAN, OSP, Vanilla-TF and our Context-TF, respectively. As it can be shown, our Context-TF model gives quite accurate predictions (in dashed blue), that in the majority of the scenarios is aligned with the ground-truth trajectory (in dashed green), given the observed short sequence (in solid dark yellow). Additionally, it can be noticed that our model can capture the non-linearity of the pedestrians’ trajectory and how it can respect the contextual information of the scene. For instance, in the second and fourth column, we can observe how resilient is the predicted trajectory from our Context-TF model that follows the non-linear ground-truth trajectory. On the other hand, we can notice how the other compared baseline models are challenged in most of the scenarios. For example, the Social-GAN, the OSP and the Vanilla-TF models in the scenario from the DUT dataset in the third column of Figure 4. We can observe how the predictions from the three models diverge away from the ground-truth trajectory, while our Context-TF model provided more confident predictions.

#### 4.2.2. Run-Time Performance

Given the fact that our proposed approach is targeted at socially aware autonomous vehicles, having a responsive model that can provide accurate future predictions about pedestrians in a timely manner is inevitable. To that end, we have evaluated the run-time performance of our proposed approach during the inference time in comparison to the aforementioned baseline approaches from the literature. Our proposed approach and all the baseline approaches were bench-marked on the same machine running on a single-core Intel Core i9-7980XE CPU with a clock rate of 2.60 GHz and an NVIDIA GeForce GTX 1080 GPU. For a single pedestrian trajectory prediction of 5 s ahead, our proposed approach ‘Context-TF’ achieved 0.025 s (i.e., 40 predictions per second). In comparison, OSP achieved only 0.03 s, while Social-GAN and MATF achieved 0.43 s and 0.84 s, respectively. As it can be noticed, our proposed approach ‘Context-TF’ has outperformed the baseline approaches in terms of run time, especially the data-driven approaches by a large margin, which makes our approach more suitable for real-time deployment.

#### 4.2.3. nuScenes Results

In our second experiment, we will be utilising the nuScenes dataset, a large-scale dataset that is unlike the DUT and inD datasets; it was captured using vehicle-based sensors similar to the ones that exist in autonomous vehicles. In our experiment, we will compare the performance of our proposed ‘Context-TF’ against three baseline models. The first one is the constant velocity model, the second one is the same Context-LSTM model reported in Table 1 and the final baseline model is one of the recent top-performing techniques on the trajectory prediction task of the nuScenes dataset, Trajectron++ [27]. Similar to our proposed approach, Trajectron++ utilise the contextual information surrounding pedestrians in a shared urban traffic environment besides the past positional information to predict their future trajectories. In Trajectron++, they represent pedestrians in the scene as graph nodes and model their interactions via graph edges, which are then fused together along with semantic map information and are fed to an encoder-decoder LSTM network. The hyper-parameters utilised for Trajectron++ are the same as the ones reported in the original paper in [27]. For a fair comparison, we have utilised the same observed trajectory δ size of 8 (i.e., 4 s) similar to Trajectron++ and the prediction horizon κ of size 6 (i.e., 3 s ahead). In Table 4, we report the results of our experiment, and similar to the DUT and inD datasets, we utilised the ADE score, but instead of the RMSE score, we used the Final Displacement Error (FDE) score just to conform to the evaluation metrics used by Trajectron++ for a fair comparison. FDE measures the Euclidean distance between the predicted final position and the ground truth final position. Furthermore, we have reported the ADE/FDE scores over four different prediction horizons (namely 1, 2, 3 and 4 s ahead). As it can be noticed from the reported ADE/FDE scores in Table 4, our proposed ‘Context-TF’ model has outperformed the closest competitor model, Trajectron++, by a large margin over the four prediction horizons, which further demonstrates the capability of our proposed approach. Furthermore, our ‘Context-TF’ model continued to outperform the LSTM-based approach similar to the reported results on the DUT and inD datasets.

## 5. Conclusions

In this work, we have introduced a novel context-augmented transformer networkbased model for the task of pedestrians’ trajectory prediction in an urban shared traffic environment. Besides their past trajectories, the proposed model also took into account the contextual information around pedestrians in the scene (i.e., interactions with other agents and the scene’s semantics information). The inclusion of such information led the model to achieve robust results on real-life datasets of pedestrians’ trajectories in urban shared traffic environments. When tested on a large-scale autonomous vehicle’s dataset of pedestrians’ trajectories, the proposed model has outperformed the compared baseline models from the literature with a large margin over short and long term prediction horizons. More specifically, our approach has improved over the SOTA approach (Trajectron++) by more than 2 cm and 3 cm over the average displacement error and the final displacement error, respectively. Furthermore, the proposed framework has demonstrated robust generalisation capabilities on unseen scenarios across different urban traffic shared spaces. Moreover, our approach has achieved superior run-time performance during the inference phase of only 0.025 s (i.e., 40 trajectory predictions per second). As a result, this would make our approach easier to deploy, as it already can achieve real-time performance, which is required for safety critical applications such as autonomous vehicles.

In our future work, we will explore other potential configurations for the input contextual feature representations, such as efficient graph neural networks or inverse reinforcement learning, where all the input features could be learned via a reward function that can capture the preference of pedestrians in a shared urban traffic environment. Consequently, we will evaluate how it might influence the performance of our contextual transformer network architecture. Additionally, in our future work we will explore the possibility of having the output predictions from our framework as an estimated probability distribution over potential positions rather than the current value-based estimation nature of our framework.

## Figures and Tables

**Table 1:** The performance of transformer-based approaches in comparison to LSTM-based approaches (w/o context input) for pedestrians’ trajectory prediction over a prediction horizon of 5 s ahead. The reported scores are shown according to the evaluation metrics (ADE/RMSE) in meters. Lower scores are better.

Dataset	DUT		inD	
**Model**	**ADE**	**RMSE**	**ADE**	**RMSE**
Vanilla-LSTM	1.52	1.06	1.38	1.07
Vanilla-TF [17]	1.21	0.95	1.07	0.96
Context-LSTM	1.16	0.95	1.03	0.96
Context-TF (ours)	**0.97**	**0.88**	**0.80**	**0.88**

**Table 2:** Ablation study on the influence of individual contextual information on DUT and inD datasets. The prediction horizon is 5 s ahead. The reported scores are shown according to the evaluation metrics (ADE/RMSE) in meters. Lower scores are better.

Dataset	DUT		inD	
**Model**	**ADE**	**RMSE**	**ADE**	**RMSE**
Context-TF${}_{POS}$	1.34	1.07	1.05	0.99
Context-TF${}_{INT}$	1.33	1.07	1.01	0.97
Context-TF${}_{SEM}$	1.41	1.08	0.99	0.94

**Table 3:** The performance of our proposed Context-TF in comparison to baseline approaches from the literature. The reported scores are shown according to the evaluation metrics (ADE/RMSE) over five different prediction horizons. Lower scores are better.

Dataset	DUT					inD				
**t (Second)**	**Linear-CV**	**Social-GAN [6]**	**MATF [35]**	**OSP [24]**	**Context-TF (Ours)**	**Linear-CV**	**Social-GAN [6]**	**MATF [35]**	**OSP [24]**	**Context-TF (Ours)**
1	0.39/0.38	0.62/0.66	0.63/0.72	**0.22**/**0.30**	0.41/0.54	0.50/0.50	0.98/1.09	0.42/0.50	0.12/0.37	**0.11**/**0.34**
2	0.84/0.82	0.86/0.96	1.22/1.40	**0.49**/**0.64**	0.65/0.69	1.10/1.13	1.58/1.79	0.86/1.03	0.37/0.83	**0.26**/**0.50**
3	1.31/1.28	1.21/1.43	1.87/2.15	0.78/1.01	**0.77**/**0.76**	1.79/1.85	2.24/2.56	1.40/1.68	0.67/1.35	**0.42**/**0.64**
4	1.81/1.75	1.67/2.02	2.58/2.97	1.09/1.37	**0.88**/**0.82**	2.57/2.64	2.96/3.39	2.00/2.40	1.02/1.92	**0.60**/**0.77**
5	2.31/2.22	2.20/2.73	3.37/3.85	1.41/1.74	**0.97**/**0.88**	3.42/3.50	3.74/4.28	2.65/3.20	1.42/2.53	**0.80**/**0.88**

**Table 4:** The performance of our proposed Context-TF approach in comparison to baseline approaches on the validation split of the nuScenes dataset [31]. The reported scores are shown according to the evaluation metrics (ADE/FDE) in centimetres. Lower scores are better.

Dataset	nuScenes			
**Model**	**@1 s**	**@2 s**	**@3 s**	**@4 s**
Linear-CV	14.08/18.78	24.56/43.86	35.93/61.61	48.17/89.88
Context-LSTM	15.44/26.23	24.32/39.30	33.36/61.18	47.39/85.66
Trajectron++ [27]	12.06/16.27	21.51/36.20	32.10/59.32	43.88/85.85
Context-TF (ours)	**11.09/14.99**	**19.91/33.62**	**30.02/55.80**	**41.39/82.36**

## Data Availability

All datasets used for training and evaluating the performance of our proposed approach are publicly available and can be accessed from [29,30,31].
